# In‐vivo‐ und Ex‐vivo‐Sonografie zur Beurteilung der Tumorränder während mikrographisch kontrollierter Chirurgie: ein vielversprechendes neues Verfahren in der Dermatoonkologie?

**DOI:** 10.1111/ddg.15827_g

**Published:** 2025-10-23

**Authors:** Diana Crisan, Ximena Wortsman, Fernando Alfageme, Karin Scharffetter‐Kochanek, Maria Crisan, Lukas Bernhard, Evelyne Tarnowietzki, Lars Alexander Schneider, Monika‐H. Schmid‐Wendtner

**Affiliations:** ^1^ Klinik für Dermatologie und Allergologie Universitätsklinikum Ulm Ulm Deutschland; ^2^ Abteilung für Dermatologie Medizinische Fakultät Pontificia Universidad Católica de Chile Santiago Chile; ^3^ Abteilung für Dermatologie Fakultät für Medizin Universidad de Chile Santiago Chile; ^4^ Institut für Diagnostische Bildgebung und Forschung der Haut und Weichteile Santiago Chile; ^5^ Abteilung für Dermatologie und Hautchirurgie University of Miami Miller School of Medicine Miami USA; ^6^ Dermatologie Hospital Universitario Puerta De Hierro Majadahonda Madrid Spanien; ^7^ Dermatologie Universität für Medizin und Pharmazie Iuliu Hațieganu Cluj‐Napoca Rumänien; ^8^ Interdisziplinäres Onkologisches Zentrum München Deutschland; ^9^ Abteilung für Dermatologie und Allergologie Ludwig‐Maximilians‐Universität München Deutschland

**Keywords:** Bildgebung, Dermatochirurgie, Hautkrebs, Mikrographisch kontrollierte Chirurgie, Nicht melanozytärer Hautkrebs, Ultraschall, dermatosurgery, imaging, micrographic controlled surgery, non‐melanoma skin cancer, skin cancer, ultrasound

## Abstract

**Hintergrund und Zielsetzung:**

Die vorliegende Untersuchung befasste sich mit der In‐vivo‐ und Ex‐vivo‐Anwendung von hochfrequentem Ultraschall (HFUS) während mikrographisch kontrollierter Chirurgie (MKC) bei nichtmelanozytärem Hautkrebs (NMSC) im Kopf‐ und Gesichtsbereich, insbesondere im Hinblick auf die perioperative Beurteilung der Resektionsränder zur potenziellen Reduktion von Operationsschritten und der Hospitalisierungsdauer der Patienten.

**Patienten und Methodik:**

Die präoperative In‐vivo‐ und Ex‐vivo‐Sonografie von 136 nichtmelanozytären Hauttumoren (NMSC) bei 111 Patienten wurde retrospektiv im Hinblick auf die Tumorrandbeurteilung während mikrographisch kontrollierter Chirurgie (MKC) durch einen erfahrenen Dermatochirurgen ausgewertet, während die Präparate unabhängig durch einen Histopathologen beurteilt wurden.

**Ergebnisse:**

Unsere Ex‐vivo‐Beurteilung der Tumorränder zeigte eine Spezifität von >98 % für das Plattenepithelkarzinom (SCC) und das Basalzellkarzinom (BCC), mit lediglich fünf falsch‐negativen und zwei falsch‐positiven Befunden im Vergleich zur Histopathologie. HFUS identifizierte tumorfreie Ränder korrekt bei 89% der untersuchten Tumoren nach der ersten Resektion sowie acht unvollständige Exzisionen (6%), bei denen eine Nachresektion durchgeführt und Tumorreste histologisch nachgewiesen wurden. Insgesamt zeigte HFUS eine sehr hohe Genauigkeit sowohl in der Detektion tumorfreier Resektionsränder als auch von Tumorresten.

**Schlussfolgerungen:**

HFUS ist eine hochpräzise Methode zur schnellen Beurteilung der Tumorränder während der MKC bei NMSC in ästhetisch besonders sensiblen Arealen. Sie hat das Potenzial, die Anzahl der Operationsschritte und die Hospitalisierungsdauer bei dermatochirurgischen Patienten signifikant zu reduzieren und damit die Qualität der Patientenversorgung zu verbessern.

## EINLEITUNG

Hochfrequenz‐Ultraschall (HFUS) ist eine moderne, nichtinvasive bildgebende Methode, die zunehmend in der Dermato‐Onko‐Chirurgie zur Visualisierung und Charakterisierung von Hauttumoren hinsichtlich Größe, Form, Beziehung zu neurovaskulären, knorpeligen oder knöchernen Strukturen sowie zur Beurteilung der Gefäßversorgung eingesetzt wird.^1^

Die durch HFUS vor der Therapie bereitgestellten Informationen sind entscheidend für die Patientenberatung und die Wahl des optimalen therapeutischen Ansatzes gemäß onkologischen Leitlinien, wobei das funktionelle und ästhetische Ergebnis für unsere Patienten erhalten bleiben soll.^2^, ^3^, ^4^ Die häufigsten Arten von nichtmelanozytärem Hautkrebs (NMSC) sind das Basalzellkarzinom (BZK) und das Plattenepithelkarzinom (PEK), wobei UV‐Bestrahlung als Hauptursache gilt. Daher ist der Kopf‐ und Gesichtsbereich eine häufige Lokalisation für deren Entwicklung.^5^ Besonders im Kopf‐ und Gesichtsbereich ist das Risiko einer unvollständigen primären Resektion höher als am Rumpf.^6^ Diese Tumoren werden häufig mit schmalen chirurgischen Resektionsrändern entfernt – entweder mittels Mohs‐ oder mikrographisch kontrollierter Chirurgie (MKC) –, um gesundes Gewebe zu schonen, die Ästhetik zu bewahren, die Funktionalität zu erhalten und den Umfang der chirurgischen Rekonstruktion zu reduzieren.^7^ In Studien konnte gezeigt werden, dass das Risiko für lokale Rezidive bei PEK und BZK signifikant niedriger ist, wenn eine MKC durchgeführt wird.^8^

Die Mohs‐Chirurgie ermöglicht die intraoperative zirkumferentielle und tiefe Tumorrandbeurteilung durch gestufte Resektionen und unmittelbare histopathologische Auswertungen. Sie stellt ein hocheffektives Verfahren für die rasche Entfernung von Hochrisiko‐Tumoren dar.^9^ Sie erfordert jedoch in der Regel mehrere chirurgische Eingriffe, ist zeitaufwendig, teuer und vom Operateur abhängig, was ihre Anwendung einschränkt. Die Slow‐Mohs‐Chirurgie oder MKC, die häufig in deutschen Zentren durchgeführt wird, bezeichnet eine gestufte chirurgische Exzision, bei der die Proben zur histopathologischen Untersuchung ins Labor geschickt werden. Die pathologischen Ergebnisse liegen in der Regel nach 1–2 Tagen vor. Bei histologisch nachgewiesenen tumorfreien Rändern (R0‐Situation) wird der Defekt verschlossen. Bei tumorinfiltrierten Rändern (R1) erfolgt eine Nachresektion und die Patienten warten auf die histologische Auswertung der Nachresektion. Je nach Tumorsubtyp sind mitunter mehrere Nachresektionen erforderlich, was die Hospitalisierungsdauer der Patienten verlängert, die Gesundheitskosten erhöht und das Risiko perioperativer Komplikationen bei den meist älteren Patienten steigert.^10^

Im Vergleich zur Dermatoskopie, konfokalen Mikroskopie (CM) und optischen Kohärenztomographie (OCT) weist HFUS bei der Beurteilung von Hauttumoren keine Einschränkung der Eindringtiefe auf, während die meisten optischen Verfahren lediglich wenige Millimeter ins Gewebe vordringen.^10^, ^11^ Präoperativer HFUS liefert dreidimensionale Echtzeitinformationen zu Tumoren, die es den Klinikern ermöglichen, ihre Exzisionen präziser zu planen, die Resektionsränder genau zu bestimmen und Rezidivraten zu senken.^12^

In unserer retrospektiven Studie haben wir die Wirksamkeit des präoperativen In‐vivo‐ und intraoperativen Ex‐vivo‐HFUS zur Identifikation von Tumorrändern während der MKC von NMSC im Kopf‐ und Gesichtsbereich vor der histologischen Beurteilung untersucht und die Ergebnisse mit den histologischen Befunden korreliert. Zudem haben wir evaluiert, inwiefern Ex‐vivo‐Ultraschall die Anzahl der chirurgischen Schritte während der MKC reduzieren kann, indem tumorfreie Resektionsränder vorhersagt werden.

## PATIENTEN UND METHODIK

Wir führten eine retrospektive Analyse der Krankenakten von 111 Patienten durch, die sich zwischen Juli 2023 und September 2024 in der dermatochirurgischen Abteilung (Klinik für Dermatologie und Allergologie, Universitätsklinikum Ulm) zur mikrographisch kontrollierten Chirurgie (MKC) von NMSC, insbesondere BZK und PEK, im Kopf‐ und Gesichtsbereich vorstellten. Zunächst wurden die Tumorresektionsränder vom Chirurgen anhand des klinischen Befundes auf der Haut eingezeichnet. Anschließend wurde eine sonografische Beurteilung der Läsion durchgeführt, bei der die Form sowie die laterale und tiefe Infiltration (mm) bewertet wurden. Die lateralen Tumorränder, wie sie im Ultraschall sichtbar waren, wurden markiert und/oder entsprechend der sonografischen Beurteilung kurz vor der Operation mit einem chirurgischen Hautmarker angepasst.

Für anspruchsvolle anatomische Bereiche wie Nasenlöcher, Concha, Fossa triangularis oder periorbitaler Bereich verwendeten wir eine große Menge Gel unter der Hockeystick‐Sonde, um hochqualitative Sonogramme ohne Artefakte zu erhalten. Zudem platzierten wir kleine Tupfer in den Nasenlöchern oder im Gehörgang, um eine Gelansammlung zu verhindern. Für die sonografische Beurteilung von hyperkeratotischen Tumoren oder Tumoren mit hämorrhagischen Krusten wurden diese vor der Untersuchung entfernt, um Artefakte zu vermeiden, die die sonografische Befundung – insbesondere die korrekte Identifizierung der tiefen Ränder – beeinträchtigen könnten. Die Tumoren wurden entsprechend der Markierung des lateralen Randes mit minimalem Sicherheitsabstand reseziert. Die sonografische Untersuchung wurde mit einem Mindray TE5 Multitouch‐Ultraschallsystem (Mindray Medical, Shenzhen) unter Verwendung einer linearen Array‐Hockey‐Stick‐Sonde (L16‐4Hs, 4–16 MHz) oder alternativ mit einem Canon Aplio A Series‐System (Canon Medical Systems) mit einer linearen Array‐Hockey‐Stick‐Sonde (17LH7, 17 MHz) durchgeführt. Bei einer Betriebsfrequenz von 15–18 MHz beträgt die axiale Auflösung typischerweise circa 100 µm, die laterale Auflösung circa 200–300 µm. Die Eindringtiefe ist bei modernen multifrequenzfähigen Hochfrequenzschallköpfen variabel einstellbar und liegt zwischen 0,1 mm und 60 mm.^1^, ^13^, ^14^

Die kompakte Bauweise der Hockey‐Stick‐Sonde ermöglicht eine besonders präzise Anwendung im Gesichtsbereich, insbesondere bei der sonografischen Untersuchung von Tumoren in der Nasen‐, Ohr‐ oder periorbitalen Region.

Für die Ex‐vivo‐Beurteilung wurde das exzidierte Präparat – analog zur In‐vivo‐Untersuchung – mit Fadenmarkierung versehen, auf einer Kompresse positioniert und großzügig mit Ultraschallgel bedeckt. Die abgedeckte Ultraschallsonde wurde sowohl in vivo als auch *ex vivo* senkrecht auf die Tumoroberfläche aufgesetzt, und es erfolgte eine zweidimensionale Evaluation (longitudinal und transversal), um die Tumorränder zu identifizieren. Diese wurden als echoarme Bereiche im Vergleich zum normalen umgebenden Gewebe identifiziert. Im Falle eines sonografischen Verdachts auf residuale Tumoranteile im Schnittrand wurde eine lokale Nachexzision in gleicher Sitzung durchgeführt. Anschließend wurden beide Proben zur pathologischen Beurteilung eingeschickt. Der Defekt nach erfolgter Tumorresektion wurde für 1–2 Tage mit einem Verband abgedeckt, während die histopathologischen Ergebnisse abgewartet wurden.

Unsere Patienten wurden nach den folgenden Einschlusskriterien ausgewählt: Verdacht/histologische Bestätigung eines BZK oder PEK, Lokalisation im Kopf‐ und Gesichtsbereich, vollständige klinische und sonografische Daten. Ausschlusskriterien umfassten oberflächliche Läsionen, die für eine nichtchirurgische Therapie qualifizierten, oder Patienten mit unvollständigen Krankenakten. Alle perioperativen sonografischen Untersuchungen wurden von demselben Operator mit umfangreicher Erfahrung in HFUS durchgeführt (D.C.). Die histologische Auswertung erfolgte im dermatohistologischen Labor der dermatologischen Klinik des Universitätsklinikums Ulm.

Alle Verfahren wurden gemäß den ethischen Standards der Deklaration von Helsinki 2013 durchgeführt, und unsere retrospektive Studie (Chart‐Review der Patientenfälle) wurde von der Medizinischen Ethikkommission der Universität Ulm genehmigt (Nr. 324/24). Die statistische Analyse erfolgte unter Verwendung von SPSS, Version 25 (IBM Corp., Armonk, NY, USA). Für die Datenauswertung wurden deskriptive Statistiken und Häufigkeiten verwendet. Kontinuierliche Daten wurden als Mittelwert mit Standardabweichung (SD) dargestellt.

## ERGEBNISSE

Wir identifizierten 136 Tumoren bei 111 Patienten (40 Männer, 71 Frauen) mit einem Durchschnittsalter von 76 ± 12,5 Jahren, bei denen In‐vivo‐ und Ex‐vivo‐Sonografien während der MKC von PEK oder BZK im Kopf‐ und Gesichtsbereich zwischen Juli 2023 und September 2024 durchgeführt wurden. Die Tumoren umfassten 83 BZK (61%), von denen 38 (48,7%) Hochrisikosubtypen waren (sklerodermiform, infiltrativ‐wachsend, mikronodulär, multizentrisch), sowie 53 PEK (39%). Die durchschnittliche Infiltrationstiefe der BZK betrug 2,5 ± 1,7 mm, die der PEK 3,9 ± 2,7 mm.

**ABBILDUNG 1:**
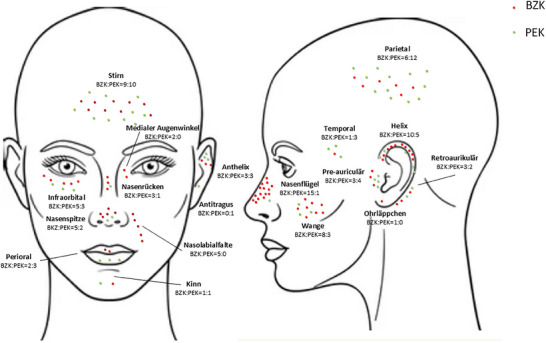
Verteilung von BZK und PEK nach anatomischer Subregion in unserer Patientenkohorte.

Die Verteilung der Tumoren nach Lokalisation und histologischem Subtyp ist in Abbildung 1 und Tabelle 1 dargestellt.

**TABELLE 1:** Verteilung der Tumoren nach histologischem Subtyp.

Tumortyp	Anzahl (n)
***Basalzellkarzinom (BZK)***	***83***
sklerodermiform	15
infiltrativ‐wachsend	10
mikronodulär	2
superfiziell/multizentrisch	11
basosquamös	2
ulzeriert	6
nodulär/pigmentiert	37
***Plattenepithelkarzinom (PEK)***	***53***
gut differenziert	14
mäßig differenziert	6
schlecht differenziert	9
ulzeriert	4
nicht klassifiziert	20

**ABBILDUNG 2:**
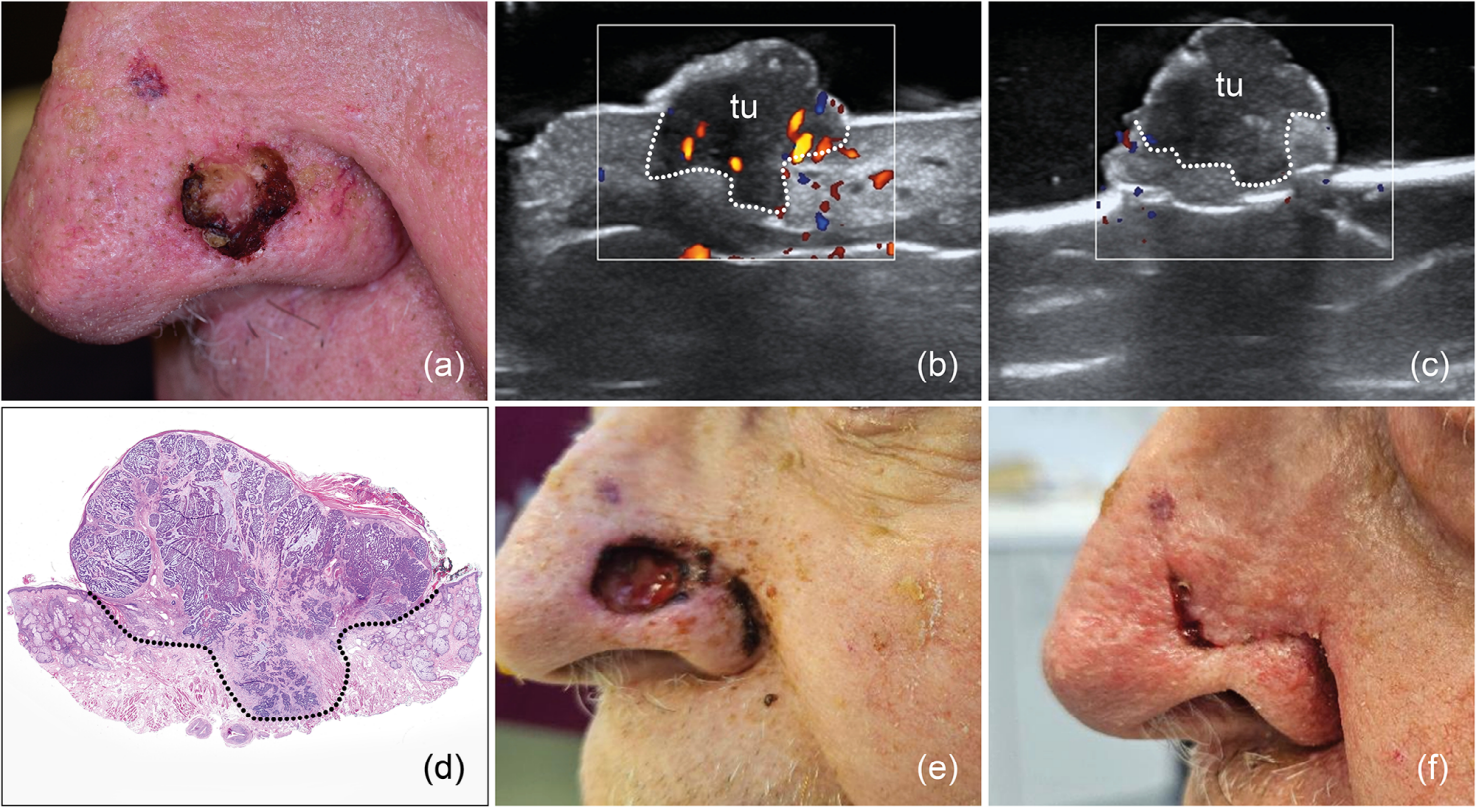
(a) Solid‐zystisches BZK des linken Nasenflügels. (b) In‐vivo‐Sonografie zeigt eine echoarme Läsion mit einigen echoreichen Punkten (Spots) und basaler Vaskularisation, die sich in der Dermis befindet und zentral eine Protrusion in das subkutane Gewebe aufweist, mit „tu“ markiert. (c) Ex‐vivo‐Sonografie zeigt den resezierten Tumor, markiert mit „tu“, mit minimalem Abstand zum basalen Exzisionsrand und freien lateralen Rändern. (d) Histologischer Aspekt des entfernten Präparats mit tumorfreien Rändern auf allen Seiten; das Aussehen des Präparats entspricht dem sonografischen Bild (Hämatoxylin‐Eosin‐Färbung [HE]). (e) Chirurgischer Defekt auf Höhe des Nasenflügels nach Tumorresektion. (f) OP‐Gebiet eine Woche nach der Defektrekonstruktion mit einem Verschiebelappen.

**ABBILDUNG 3:**
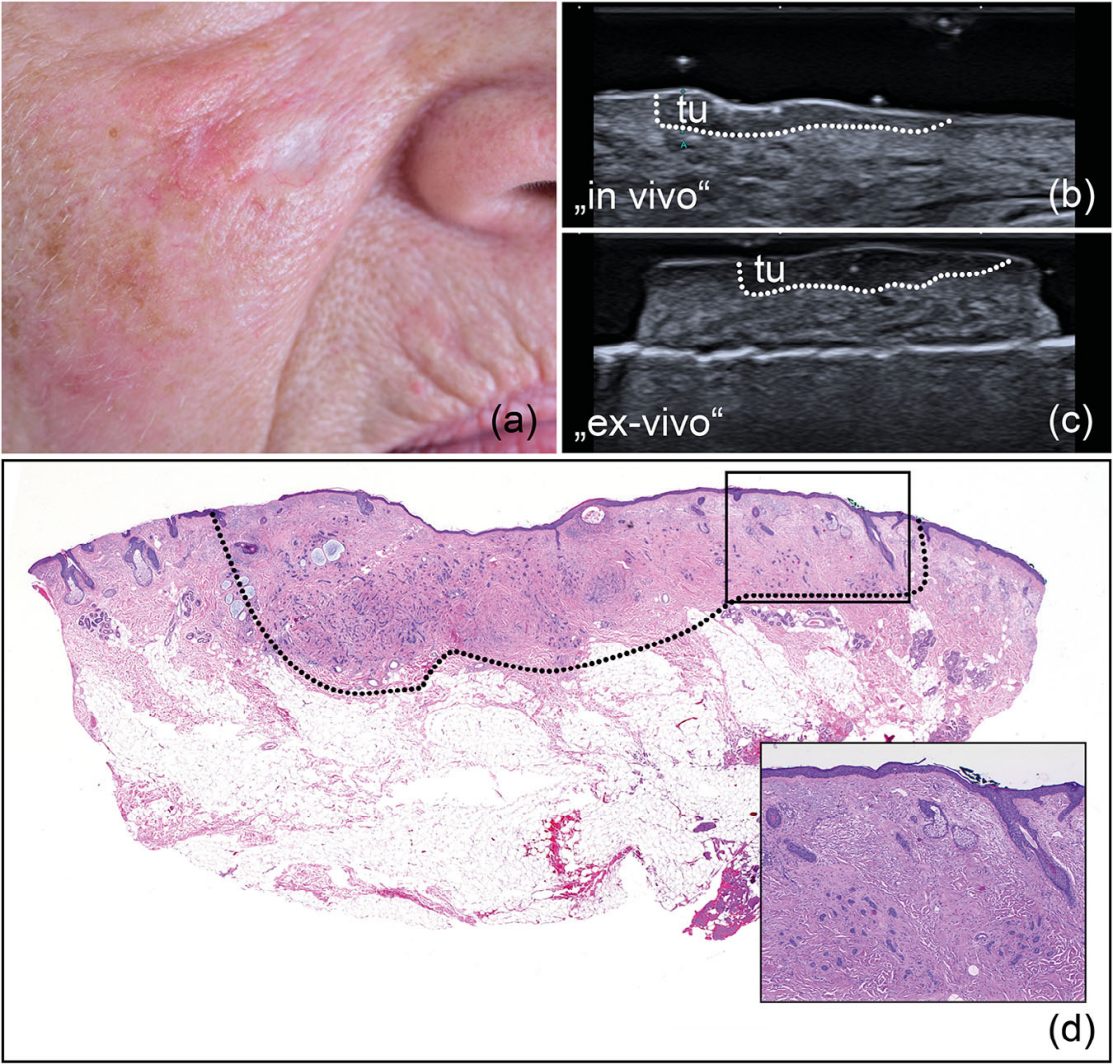
(a) Klinisches Bild eines weißlichen, unscharf begrenzten sklerodermiformen BZK an der rechten Wange. (b) In‐vivo‐Sonografie zeigt eine echoarme Läsion mit zahlreichen echoreichen Spots in der Dermis, markiert mit „tu“. (c) Ex‐vivo‐Sonografie des resezierten Tumors, markiert mit „tu“, mit Sicherheitsabstand sowohl zum basalen als auch zum lateralen Resektionsrand. (d) Histologischer Aspekt des entfernten Präparats mit tumorfreien Rändern auf allen Seiten. Das Aussehen des Präparats entspricht dem sonografischen Bild. Inset zeigt histologische Vergrößerung (× 40) mit einzelnen Tumorzellnestern am lateralen Rand der Läsion (Hämatoxylin‐Eosin‐Färbung).

**ABBILDUNG 4:**
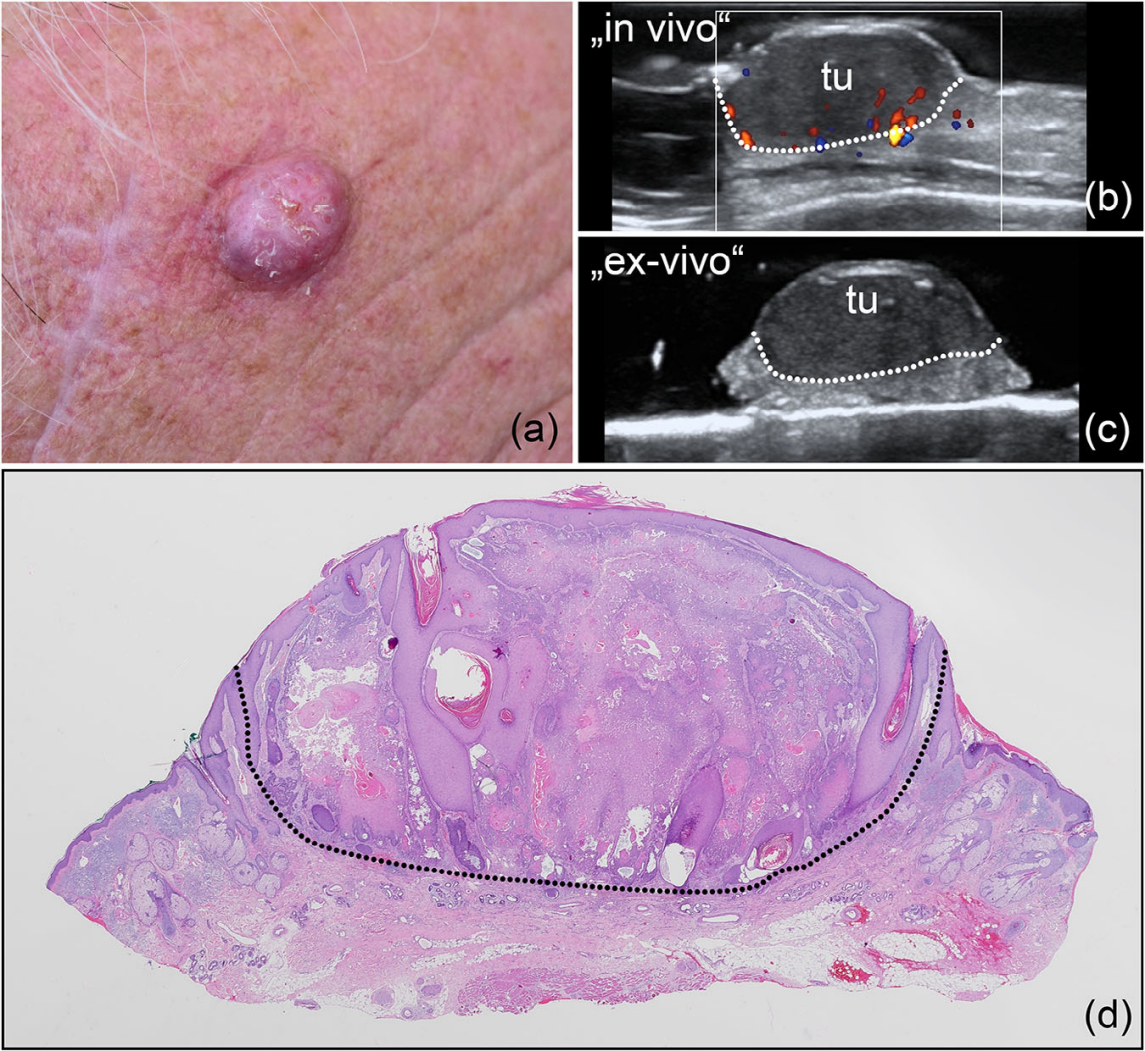
(a) Klinisches Bild eines erythematösen Knotens in der rechten Stirnregion, vereinbar mit einem PEK. (b) In‐vivo‐Sonografie zeigt eine echoarme Läsion in Dermis und Subkutis, markiert mit „tu“, mit verstärkter basaler Vaskularisation, dargestellt im Farb‐Doppler. (c) Ex‐vivo‐Sonografie des resezierten Tumors, markiert mit „tu“, mit tumorfreien Rändern lateral und in der Tiefe. (d) Histologischer Aspekt des entfernten Präparats, bestätigend ein akantholytisch‐zystisches PEK, vollständig reseziert (Hämatoxylin‐Eosin‐Färbung).

**ABBILDUNG 5:**
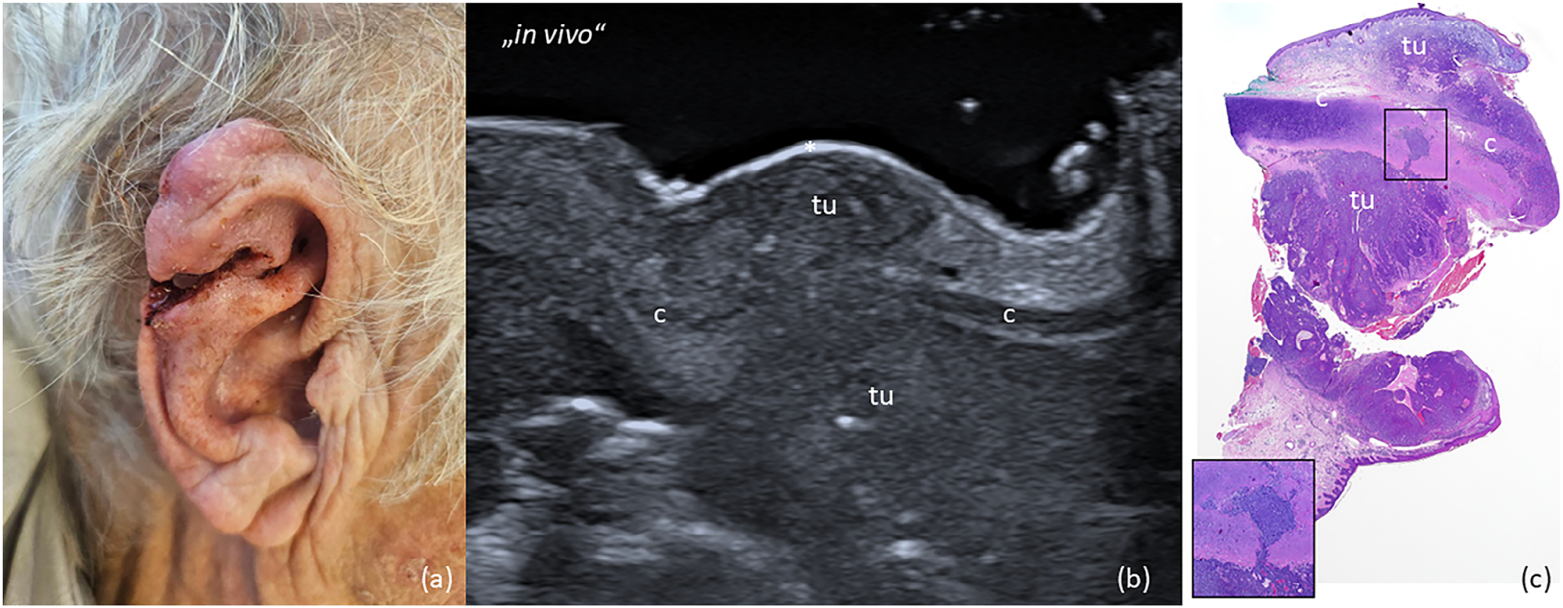
(a) Klinisches Bild eines ulzerierten BZK im rechten Ohrbereich. (b) In‐vivo‐Sonografie zeigt eine echoarme Läsion, markiert mit „tu“, die den Knorpel infiltriert, markiert mit „*“. (c) Histologischer Aspekt des entfernten Präparats, bestätigt eine lokalisierte Knorpelinfiltration, dargestellt im Inset (Hämatoxylin‐Eosin‐Färbung, Originalvergrößerung × 40).

**ABBILDUNG 6:**
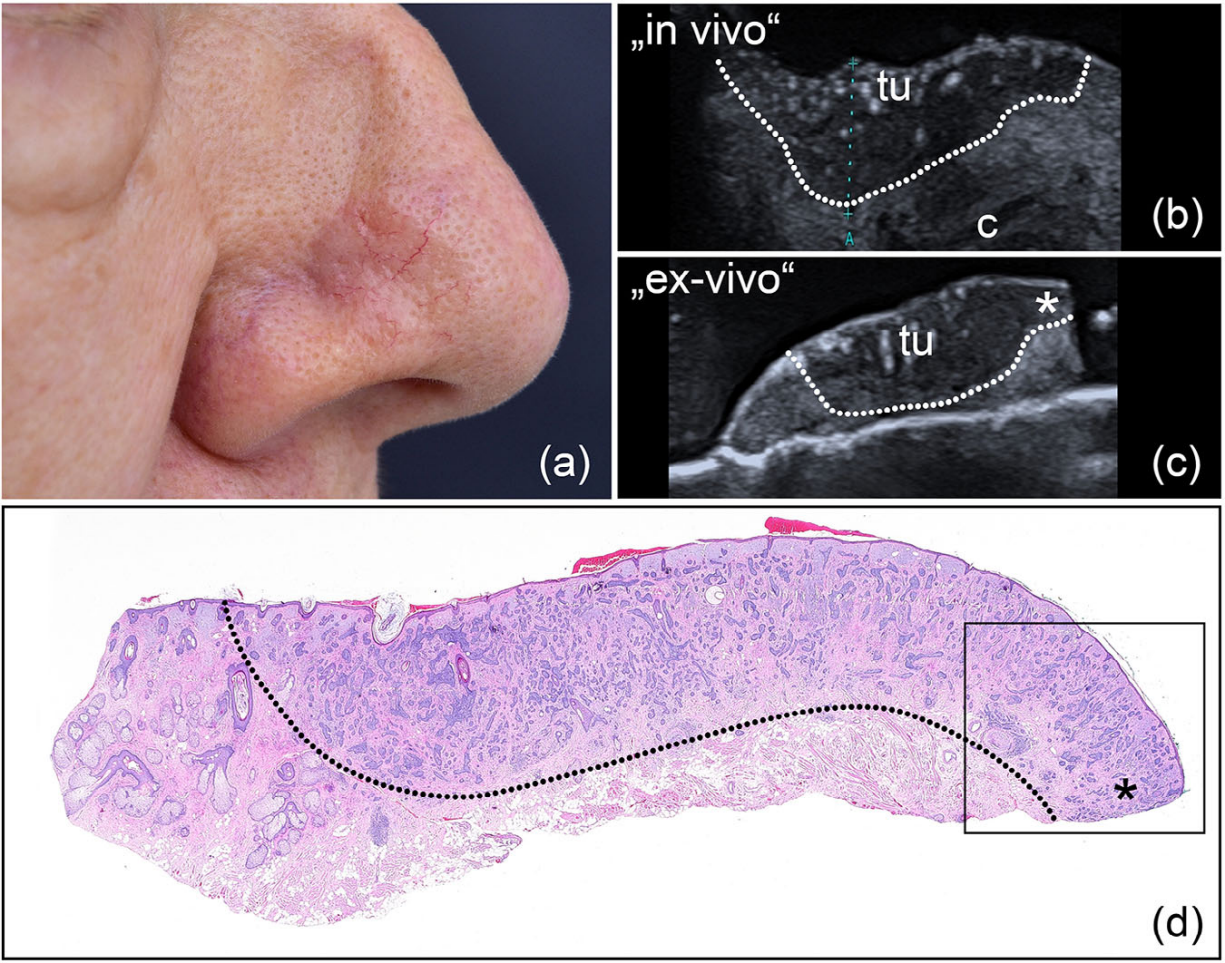
(a) Klinisches Bild eines sklerodermiformen BZK an der rechten Nasenflügelbasis. (b) In‐vivo‐Sonografie zeigt eine echoarme Läsion in Dermis und Subkutis mit mehreren echoreichen Spots, vereinbar mit einem Hochrisiko‐Subtyp, markiert mit „tu“; der Knorpel (markiert mit „c“) ist nicht vom Tumor infiltriert. (c) Ex‐vivo‐Sonografie des resezierten Tumors, markiert mit „tu“, zeigt tumorfreie Ränder auf einer Seite und in der Tiefe, jedoch Verdacht auf Tumorrest auf der anderen Seite, markiert mit „*“. (d) Histologischer Aspekt des entfernten Präparats, bestätigt eine Tumorinfiltration an einem lateralen Rand, markiert mit „*“ (Hämatoxylin‐Eosin‐Färbung).

Mit der präoperativen In‐vivo‐ und intraoperativen Ex‐vivo‐HFUS zur Tumorrandbestimmung wurden 121 (89%) der primären Tumorresektionen als tumorfrei (R0) evaluiert. Diese Ergebnisse wurden histologisch bestätigt (Abbildungen 2, 3, 4). Darüber hinaus identifizierte die präoperative Sonografie in allen untersuchten Fällen von PEK und BZK korrekt eine Infiltration von Muskel‐ oder Knorpelgewebe (5 Tumoren), wodurch eine vollständige Resektion in einem operativen Schritt ermöglicht wurde (Abbildung 5). Sechs BZK, überwiegend Hochrisikosubtypen (1 sklerodermiform, 2 infiltrativ‐wachsend, 1 multizentrisch, 1 mikronodulär, 1 solid) wurden sonografisch als R1 entweder zum seitlichen oder tiefen Rand bewertet und nachreseziert; Tumorreste wurden in allen Fällen histologisch nachgewiesen (Abbildung 6). Ein Patient mit einem soliden BZK an der Stirn zeigte seitlich oberflächliche Tumorreste (TD 0,3 mm). Er entschied sich jedoch gegen eine Nachresektion und zugunsten einer lokalen Therapie und Nachsorge. Ein mikronoduläres BZK der Nasenflügel wurde in der Ex‐vivo‐Sonografie als nicht sicher R0 betrachtet, und eine lokale Nachresektion wurde durchgeführt. Histologisch konnten keine Tumorreste gefunden werden.

In vier BZK‐Fällen (3 sklerodermiform – Helix, parietal, 1 basosquamös – Helix) zeigte die Ex‐vivo‐Sonografie tumorfreie Ränder, während die histologische Auswertung eine R1‐Situation nachwies. Beim basosquamösen Karzinom der Helix zeigte die Histologie einzelne Tumorzellen und eine perineurale Infiltration entlang des Knorpels, die bei der HFUS übersehen wurden. Zwei sklerodermiforme BZK der Helix wiesen histologisch tiefe R1‐Ränder auf; daher wurden entsprechende Nachresektionen durchgeführt; es konnten allerdings keine Tumorzellen im oder um den Knorpel herum identifiziert werden. Ein sklerodermiformes BZK im parietalen Bereich wurde sonografisch als R0 evaluiert und die Histologie zeigte einen lateralen R1‐Rand. Jedoch war die Nachresektion auf dieser Ebene tumorfrei. Die übrigen 72 untersuchten BZK zeigten in der Sonografie und Histologie tumorfreie Resektionsränder.

Drei PEK wurden in der Ex‐vivo‐Sonografie als R1 zur Tiefe beurteilt und nachreseziert. Zwei dieser Tumoren (1 ulzeriert, 1 schlecht differenziert) wiesen histologisch tiefpositive Resektionsränder auf, und in der Nachresektion wurden Tumorreste identifiziert. Bei dem dritten, mittelgradig differenzierten, parietalen PEK sagte die Ex‐vivo‐Sonografie die Notwendigkeit einer Nachresektion voraus, jedoch wurde histologisch kein residualer Tumor nachgewiesen.

Ein schlecht differenziertes PEK an der Nasenspitze wurde sonografisch als R0 evaluiert, während die Histologie eine R1‐Situation zeigte. Es waren tatsächlich zwei weitere Nachresektionen erforderlich, um tumorfreie Schnittränder zu erreichen. Die Resektionsränder der übrigen 49 exzidierten PEK wurden in der Sonografie und Histologie als tumorfrei beurteilt. Die Übereinstimmung zwischen den Ex‐vivo‐ und histologischen Befunden sowie die Sensitivität, Spezifität und diagnostische Genauigkeit der Ex‐vivo‐HFUS sind in Tabelle 2 dargestellt.

**TABELLE 2:** Sensitivität, Spezifität und Übereinstimmung der diagnostischen Genauigkeit zwischen den sonografischen und histologischen Befunden in unserer Patientenkohorte mit BZK und PEK.

BZK	Histologie R1	Histologie R0	Gesamt
Ultraschall – R1	6	1	7
Ultraschall – R0	4	72	76
Gesamt	10	73	83
Sensitivität	60%		
Spezifizität	98,6%		
Genauigkeit	93,98%		

PEK	Histologie R1	Histologie R0	Gesamt
Ultraschall – R1	2	1	3
Ultraschall – R0	1	49	50
Gesamt	3	50	53
Sensitivität	66,6%		
Spezifizität	98%		
Genauigkeit	96,2%		

## DISKUSSION

Die Mohs‐Chirurgie und die MKC sind präzise und schnelle Verfahren zur Erzielung tumorfreier Resektionsränder, insbesondere bei Hochrisiko‐Nichtmelanomhautkrebs in ästhetisch sensiblen Bereichen, in denen eine gewebeschonende Chirurgie für eine optimale Defektrekonstruktion unerlässlich ist.^15^ Während der MKC sind jedoch die Anzahl der erforderlichen chirurgischen Schritte, die endgültige Defektgröße und das Behandlungsergebnis ohne ein zuverlässiges Instrument zur präoperativen Bestimmung der Tumorränder nicht vorhersagbar.^16^

Die konfokale Mikroskopie wird zunehmend in der klinischen Praxis zur seitlichen Randbeurteilung von BZK und Lentigo maligna eingesetzt und auch *ex vivo* auf frisch exzidierten Präparaten für diese Indikation angewendet.^10^, ^16^ Die CM ermöglicht zwar die Identifizierung von BZK‐Nestern jenseits der präoperativen Randmarkierung, ist jedoch aufgrund der geringen Laserdurchdringungstiefe (200 µm) nur für oberflächliche Läsionen geeignet. Das bedeutet, dass sie tiefere Ränder, die bei vielen Hochrisikotumoren entscheidend sind, nicht beurteilen kann.^17^ Auch die optische Kohärenztomografie, die Infrarotlicht verwendet, kann Querschnittsbilder von Hauttumoren erzeugen und erreicht eine Eindringtiefe von bis zu 1,5 mm. Studien haben gezeigt, dass OCT die Sensitivität und Spezifität bei der Diagnose von BZK verbessert und in 80–100% der Fälle die Tumorausdehnung über die klinischen Ränder hinaus vorhersagen kann.^18^, ^19^

Die *Line‐field confocal optical coherence tomography* (LC‐OCT) ist eine neue, vielversprechende Methode mit einer Eindringtiefe von 500 µm und einer Auflösung von 1,1–1,3 µm. Sie ermöglicht die Darstellung eines vertikalen Bildes, das einem histologischen Schnitt ähnelt, eines horizontalen Bildes, das mit der Reflektanz‐Konfokalmikroskopie vergleichbar ist und zelluläre Auflösung bietet, sowie eine dreidimensionale Ansicht der untersuchten Läsion.^20^ Die LC‐OCT wird zunehmend und erfolgreich für die frühzeitige In‐vivo‐Diagnose und therapeutische Nachbeobachtung vieler Hauterkrankungen, einschließlich Hauttumoren, eingesetzt.^21^

Diese bildgebende Methode wurde zudem auch im Ex‐vivo‐Setting als hilfreich für die Beurteilung der lateralen Tumorränder beschrieben. Allerdings reicht die Eindringtiefe lediglich bis zur mittleren Dermis, was die Beurteilung größerer Tumoren und der tiefen Ränder erschwert. Dennoch gibt es gute Daten zur LC‐OCT bei Hochrisiko‐BZK‐Patienten, die mittels Mohs‐Chirurgie operiert wurden. In dieser Patientengruppe konnte die *ex vivo* angewandte Methode durch eine Beurteilung der lateralen Tumorränder die durchschnittliche Anzahl der chirurgischen Schritte im Vergleich zur Kontrollgruppe signifikant reduzieren.^22^

Hochfrequenz‐Ultraschall (> 15 MHz) kann im Gegensatz dazu eine sehr zuverlässige perioperative Beurteilung und Charakterisierung von Tumoren in allen Dimensionen ermöglichen – ohne die Einschränkung der Eindringtiefe.^23^, ^24^, ^25^, ^26^, ^27^ Die Methode ermöglicht die präzise präoperative Identifikation von Tumorrändern und trägt zu einer verbesserten Tumorabgrenzung bei.^2^ In unserer Analyse erhöhte die präoperative Sonografie zur Markierung der Tumorränder die Anzahl der vollständigen Resektionen nach nur einem chirurgischen Schritt (93% der Läsionen) signifikant. Darüber hinaus identifizierte HFUS im präoperativen Setting korrekt eine Infiltration von Muskel‐ oder Knorpelgewebe (2 PEK und 3 BZK). Dies ermöglichte die direkte Resektion des infiltrierten Areals, reduzierte das Vorgehen um mindestens einen chirurgischen Schritt oder führte sogar zu einer Anpassung der Therapie, beispielsweise der Entscheidung für eine Radiotherapie anstelle einer Operation.

Eine Einschränkung von Hochfrequenz‐Ultraschall (HFUS) besteht in der begrenzten Differenzierung zwischen verschiedenen melanozytären Läsionen, wie sie beispielsweise durch LC‐OCT ermöglicht wird, da HFUS kein Pigment erkennen kann. Dennoch kann HFUS bei pigmentierten Läsionen, wie dem malignen Melanom (MM), die Tumorinfiltrationstiefe präzise bestimmen und ein lokoregionales Staging zum Zeitpunkt der Diagnose ermöglichen. Die präoperative Beurteilung der Tumorinfiltrationstiefe bei MM zum Zeitpunkt der Diagnose mittels Sonografie kann dabei helfen, die Indikation für eine Sentinel‐Lymphknoten‐Biopsie sowie den erforderlichen Sicherheitsabstand festzulegen.^23^

Pasquali et al. berichteten erstmals über die Verwendung eines 22‐MHz‐Hochfrequenz‐Ultraschallgeräts (Taberna ProMedicum, Lüneburg, Deutschland) zur Ex‐vivo‐Tumorrandbestimmung. Die untersuchten Läsionen durften jedoch 13 mm Länge oder 8 mm Tiefe nicht überschreiten. Dies waren die maximalen Dimensionen, die mit dem Gerät bewertet werden konnten. Neunundsiebzig von 100 Läsionen waren BZK, überwiegend nodulär, 16 waren benigne Läsionen (Nävi, Dermatofibrome), und der Rest waren PEK. In 81 von 84 Tumoren bestimmte HFUS die Tumorränder korrekt, übereinstimmend mit den histologischen Befunden. Außerdem konnte abgeleitet werden, dass Ex‐vivo‐Sonografie als perioperatives Werkzeug das Vertrauen der Chirurgen in die korrekte Identifizierung der Resektionsränder erhöhen könnte.^28^ In unserer Kohorte hatten wir signifikant größere Tumoren, die wir mithilfe von HFUS ohne Größenlimitierung umfassend charakterisieren konnten.

In einer weiteren Studie an 65 Patienten mit BZK im Gesicht, auf der Kopfhaut sowie am Rumpf und an den Extremitäten, die mit einem Sicherheitsabstand von 2–3 mm operiert wurden, zeigte die Ex‐vivo‐Sonografie (10–18 MHz) eine Spezifität von 91,6 % bei der korrekten Identifizierung der Tumorränder.^29^

**ABBILDUNG 7:**
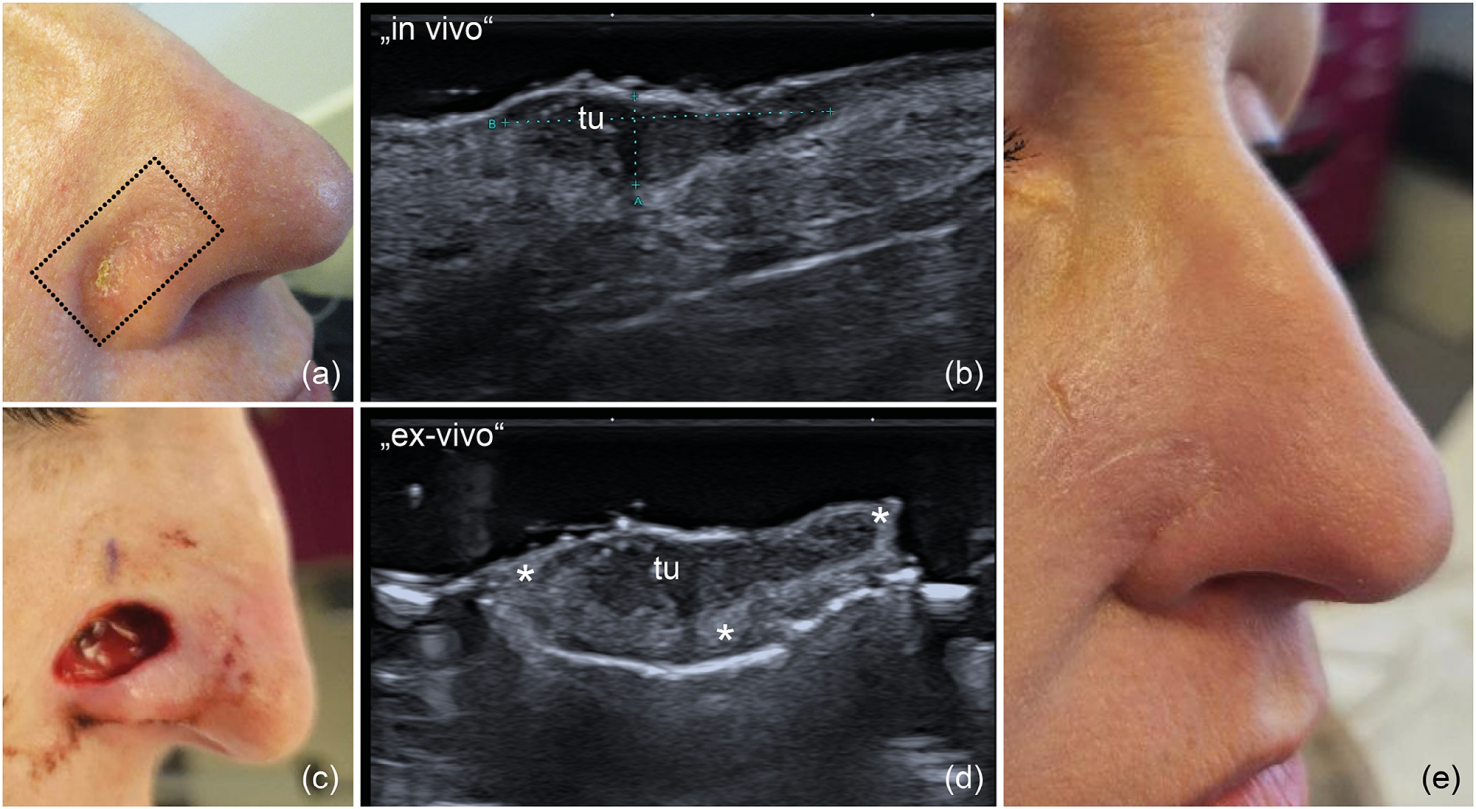
(a) Klinisches Bild eines infiltrativen BZK am rechten Nasenflügel; das Rechteck markiert die Platzierung der Ultraschall‐Sonde Sonografie‐Sonde. (b) In‐vivo‐Sonografie zeigt eine echoarme Läsion mit echoreichen Spots, lokalisiert in der Dermis und bis in das subkutane Gewebe reichend. (c) Ex‐vivo‐Sonografie des resezierten Tumors zeigt Sicherheitsabstände in der Tiefe und zu beiden Seiten, markiert mit „*“. (d) Klinisches Bild des chirurgischen Defekts nach Tumorresektion. (e) OP‐Gebiet 4 Wochen nach Defektrekonstruktion mit einem Rhomboid‐Lappen.

Die Ex‐vivo‐Beurteilung der Tumorränder in unserer Studie zeigte eine Spezifität von ≥ 98% sowohl für PEK als auch BZK, mit nur fünf falsch‐negativen und zwei falsch‐positiven Ergebnissen, während die Sensitivität bei 60% für BZK und 66% für PEK lag. Wir identifizierten in 89% der untersuchten Läsionen nach der ersten Resektion korrekterweise tumorfreie Ränder, basierend auf der präoperativen sonografischen Markierung und der Ex‐vivo‐Sonografie zur Randbeurteilung. In acht R1‐Situationen (6%) wurde eine erneute Exzision durchgeführt, die das Vorhandensein von Tumorresten bestätigte (Abbildung 7). Insgesamt wies der HFUS in unserer Kohorte eine sehr hohe diagnostische Zuverlässigkeit bei der Detektion sowohl tumorfreier Ränder als auch von Tumorresten auf (Tabelle 2).

In vier BZK‐Fällen, bei denen die Ex‐vivo‐Sonografie eine vollständige Resektion anzeigte, während die Histologie eine R1‐Situation zeigte, handelte es sich in einem Fall um ein basosquamöses Karzinom mit perineuraler Infiltration, das durch Ultraschall tatsächlich übersehen wurde. In zwei weiteren Fällen von sklerodermiformen BZK der Helix ergab die Histologie eine inkomplette tiefe Resektion; die partielle Exzision des darunterliegenden Knorpels bestätigte jedoch keinen Tumornachweis. In beiden Fällen war in der präoperativen HFUS eine intakte Knorpellinie unterhalb der Läsion erkennbar, weshalb die Resektion bis zum Knorpel durchgeführt wurde. Vermutlich stellt der Knorpel eine Barriere für das Tumorwachstum dar, da BZK häufig zunächst entlang des Knorpels wachsen, bevor sie diesen infiltrieren. In solchen Fällen kann die präoperative Sonografie wertvolle Informationen liefern, die – in Verbindung mit histologischen Befunden – helfen können, ausgedehnte Knorpelresektionen, komplexe Rekonstruktionen und gegebenenfalls auch Knorpeltransplantationen zu vermeiden. Ein sklerodermiformes BZK in der Parietalregion wurde histologisch als R1 eingestuft und nachreseziert; es konnte jedoch kein verbliebener Tumor nachgewiesen werden. Dies könnte damit erklärt werden, dass die histologische Beurteilung auf einem zuvor geschnittenen und präparierten Tumorabschnitt basiert. In einigen Fällen kann die histologische Beurteilung – abhängig von der Schnittführung – auch falsch‐positive Ergebnisse liefern.

Für ein schlecht differenziertes PEK an der Nasenspitze, das in der Ex‐vivo‐Sonografie fälschlicherweise als R0 eingestuft wurde, zeigte die Histologie eine R1‐Situation, sodass der Patient zwei weitere Re‐Exzisionen benötigte. Dies war vermutlich darauf zurückzuführen, dass der Tumor sehr oberflächlich an den lateralen Schnitträndern lag und sich in einem stark lichtexponierten Bereich mit ausgeprägter aktinischer Elastose befand. Diese ist im Ultraschall manchmal schwer von Tumorresten zu unterscheiden, da auch sie als echoarme Bandstruktur in der Dermis erscheint.^30^ Für das mäßig differenzierte PEK im Parietalbereich, bei dem die Ex‐vivo‐Sonografie eine potenzielle tiefe R1‐Situation anzeigte, ergab die Re‐Exzision keinen Tumornachweis. Der Eingriff hätte demnach vermieden werden können. Hochrisiko‐PEK sind in der Regel unregelmäßig konfiguriert, haben eine gemischte Echogenität und zeigen normalerweise periläsionale Entzündungsinfiltrate, was es manchmal erschwert, die Ränder korrekt zu beurteilen. Darüber hinaus kann die tiefere Tumorrandbestimmung schwierig sein, wenn die Hyperkeratosen von der Oberfläche der PEK nicht entfernt werden, da sonografische Artefakte auftreten können.

Die Anwendung von In‐vivo‐ und Ex‐vivo‐Sonografie verringerte die Anzahl der MKC‐Schritte bei mindestens acht unserer Patienten, die in einem chirurgischen Schritt nachreseziert wurden, wodurch insgesamt acht zusätzliche Hospitalisierungstage eingespart werden konnten. Wenn wir die 121 primären R0‐Resektionen betrachten, bei denen die Ex‐vivo‐Sonografie eine R0‐Situation anzeigte und dies histologisch bestätigt wurde, ergibt sich ein Potenzial zur Reduktion von mindestens 129 zusätzlichen Hospitalisierungstagen. Angesichts des exponentiellen Anstiegs von NMSC in letzter Zeit hätten diese zusätzlichen Tage für andere Patienten genutzt werden können, wodurch die Wartezeiten insgesamt verkürzt und die Gesundheitsressourcen effizienter eingesetzt worden wären.^31^ Andererseits empfahl die Sonografie in zwei R0‐Fällen eine Re‐Exzision, was zu größeren chirurgischen Defekten hätte führen können, die möglicherweise komplexere Rekonstruktionen erforderlich gemacht hätten.

Die Mohs‐Chirurgie ist, zumindest in den USA, sehr kostenintensiv; im Jahr 2017 beliefen sich die von Medicare erstatteten Ausgaben für Mohs‐Chirurgie auf 537 Millionen US‐Dollar – ein deutlicher Anstieg im Vergleich zu 2014. Da die Inzidenz von NMSC weiter steigt, bleibt der Bedarf an Mohs‐Chirurgie bestehen. Daher sind Ansätze erforderlich, um nicht nur die Verfahrenskosten zu senken, sondern auch die Anzahl der chirurgischen Schritte zu begrenzen. Schätzungen zufolge würde eine Reduktion der chirurgischen Schritte um nur 10% pro Patient jährlich etwa 36 Millionen US‐Dollar einsparen.^18^, ^32^

In unserer Analyse wurden ausschließlich Tumoren des Kopf‐ und Gesichtsbereichs berücksichtigt. Von insgesamt 136 Läsionen wurden 38 BZK und neun PEK aufgrund ihres histologischen Subtyps als Hochrisikotumoren eingestuft. Der Anteil wäre noch höher, wenn die Lokalisation in der H‐Zone betrachtet wird, wo die MKC die Therapie der Wahl darstellt und der Bedarf, die Anzahl der chirurgischen Eingriffe zu reduzieren, bislang nicht erfüllt werden kann. Wir konnten zeigen, dass die Ex‐vivo‐HFUS selbst bei infiltrativen, sklerosierenden BZK und niedrig differenzierten PEK die Tumorränder korrekt identifizieren und gegebenenfalls die Notwendigkeit einer sofortigen Nachresektion erkennen kann. Für die OCT ist die Ex‐vivo‐Randbeurteilung bei nodulären BZK am zuverlässigsten, während sie bei infiltrativen oder sklerosierenden Tumoren oft nicht mit den histologischen Befunden übereinstimmt.^18^, ^33^ Die konfokale Mikroskopie kann ebenfalls zur Beurteilung der Tumorränder *ex vivo* eingesetzt werden; jedoch muss das Gewebe hierfür fixiert, gefärbt, gescannt und von einem Pathologen beurteilt werden, was im Vergleich zur Sonografie mehr Zeit und Ressourcen erfordert.^10^

Hochfrequenz‐Ultraschall ist hingegen weit verbreitet und ermöglicht eine sehr schnelle Beurteilung der Tumorränder, sowohl in vivo als auch *ex vivo*. Es bleibt die einzige bildgebende Technik, welche die Tumortiefe ohne Einschränkungen der Eindringtiefe bestimmen, den Primärtumor in allen Achsen charakterisieren und bei Bedarf ein lokoregionäres Staging durchführen kann. Es konnte zudem gezeigt werden, dass bei Frequenzen ≥ 15 MHz eine höhere räumliche Auflösung als bei PET‐CT und MRT erreicht wird.^34^

**ABBILDUNG 8:**
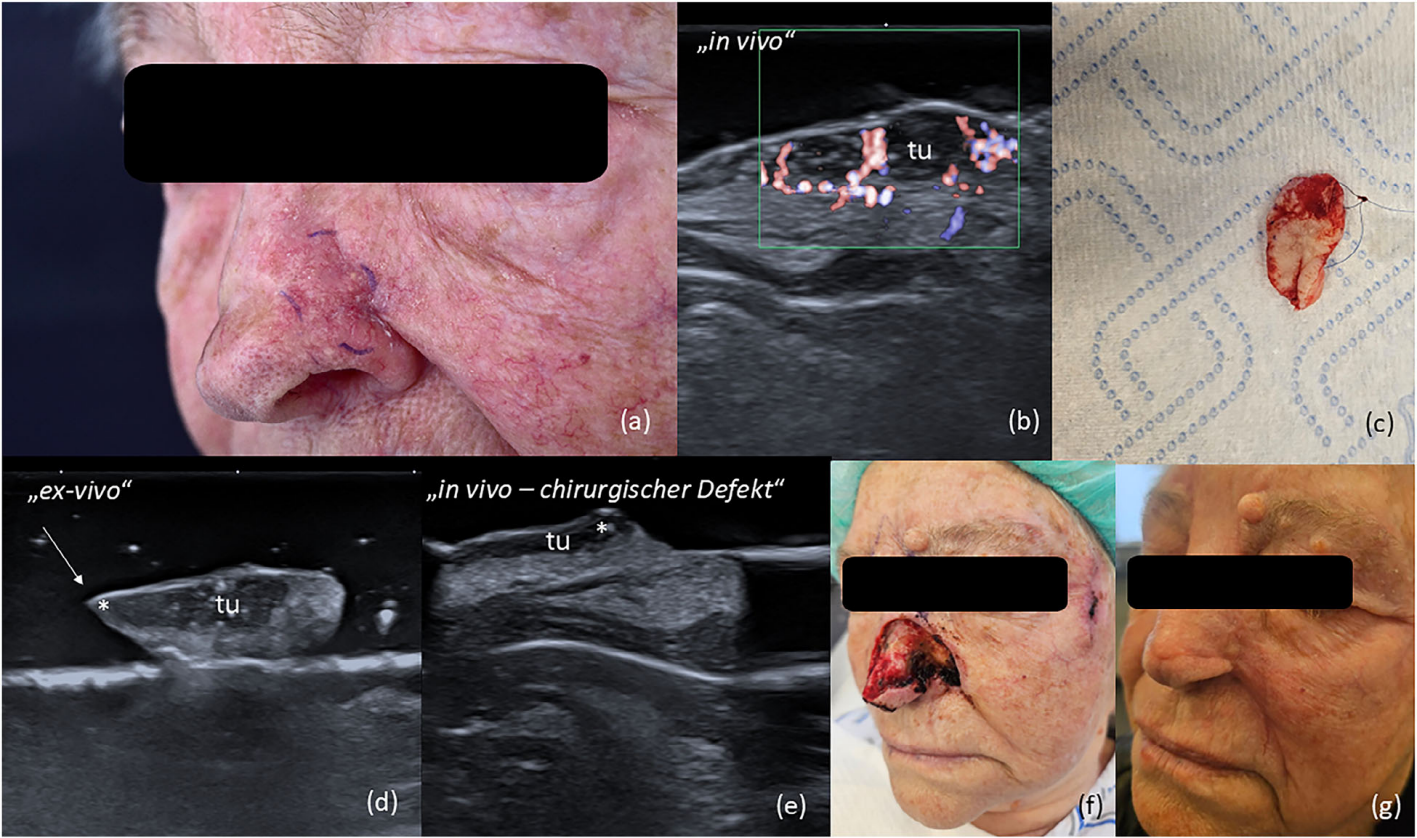
(a) Klinisches Bild eines sklerodermiformen BZK am linken Nasenflügel. (b) In‐vivo‐Sonografie zeigt eine echoarme Läsion mit echoreichen Spots und verstärkter Vaskularisation, lokalisiert in Dermis und Subkutis, markiert mit „tu“. (c) Präparat nach der ersten Exzision, vorbereitet für die Ex‐vivo‐Sonografie. (d) Ex‐vivo‐Sonografie des resezierten Tumors zeigt eine Tumorinfiltration an einer Seite, markiert mit „*“. (e) In‐vivo‐Sonografie des chirurgischen Defekts zeigt einen Tumorrest an einem Resektionsrand, markiert mit „*“. (f) Klinisches Bild des chirurgischen Defekts nach vollständiger Tumorresektion. (g) OP‐Gebiet 6 Wochen nach Defektrekonstruktion mit einem Verschiebelappen aus Glabella und Nasolabialfalte.

Die Ex‐vivo‐HFUS kann am frisch resezierten Präparat vor der histopathologischen Auswertung als Bedside‐Verfahren eingesetzt werden, um Tumorränder zu bestimmen und unmittelbar eine therapeutische Entscheidung zu treffen. Dies könnte den Patienten zusätzliche chirurgische Eingriffe und perioperative Schmerzen, die mit der Infiltrationsanästhesie verbunden sind, ersparen und eine schnellere Defektdeckung ermöglichen. Zudem könnte in Fällen, in denen die Ex‐vivo‐Sonografie eindeutig tumorfreie Ränder anzeigt, auf die histologische Auswertung verzichtet werden, sodass der chirurgische Defekt unmittelbar rekonstruiert werden kann. Eine weitere mögliche Anwendung während der MKC könnte das Durchführen einer In‐vivo‐Sonografie des chirurgischen Defekts in Fällen von R1‐Rändern sein, um das verbleibende Tumorgewebe zu vermessen und so eine lokalisierte, gewebeschonende Nachexzision zu ermöglichen – insbesondere in ästhetisch sensiblen Arealen (Abbildung 8).

Eine Einschränkung der HFUS besteht in der Schwierigkeit, oberflächliche laterale Tumorränder in lichtexponierten Arealen präzise zu identifizieren. Dies ist auf das *subepidermale low echogenicity band* (SLEB) zurückzuführen, eine echoarme Zone in der oberen Dermis, die infolge chronischer Lichtschädigung entsteht. Zusätzlich kann eine oberflächliche perineurale Infiltration, durch einzelne Zellen oder kleine Tumorzellnester – wie in unserem Fall –, im Ultraschall übersehen werden. In solchen Situationen sollten andere bildgebende Verfahren mit einer höheren Auflösung der oberen Hautschichten, wie die konfokale Mikroskopie oder die LC‐OCT, in Betracht gezogen werden.^35^ Eine weitere Möglichkeit ist die Ergänzung der Untersuchung durch ultrahochfrequenten Ultraschall (UHFUS) mit Frequenzen von 50 bis 70 MHz, der eine bessere Erkennung oberflächlicher Tumoren mit einer axialen räumlichen Auflösung von bis zu 30 µm ermöglicht.^36^ Die Anwendung bei BZK zeigte eine Intraklassen‐Korrelation von ≥ 0,8 zwischen Ultraschall‐ und histologischen Befunden und wurde auch bei oberflächlichen Plattenepithelkarzinomen erfolgreich eingesetzt.^5^, ^37^, ^38^ Diese Geräte sind jedoch weltweit nur in wenigen Zentren verfügbar, während HFUS weit verbreitet ist.

In seltenen unklaren Fällen – insbesondere bei Hochrisiko‐Tumoren im Gesichtsbereich, die häufig lokale Lappenplastiken zur Rekonstruktion erfordern – sollten Patienten darüber informiert werden, dass die Ex‐vivo‐Sonografie fälschlicherweise tumorfreie Ränder anzeigen kann (beispielsweise bei perineuraler Infiltration oder oberflächlichen Tumorzellnestern in lichtexponierten Arealen). Daher sollte die histologische Bestätigung vor dem Defektverschluss abgewartet werden. Bei Fällen die primär verschlossen werden können ist eine lokale Re‐Exzision in der Regel kein Problem. Allerdings könnte eine Nachresektion nach erfolgter Lappenplastik eine Herausforderung darstellen, da hier die korrekte Identifizierung der R1‐Areale durch die Rotation/Transposition/Verschiebung des Gewebes erschwert wird. Für Fälle, in denen die Ex‐vivo‐Sonografie die Tumorränder eindeutig identifiziert, kann der Defektverschluss vorab mit dem Patienten besprochen werden. Es ist jedoch wichtig zu betonen, dass die Durchführung von HFUS gut ausgebildete Dermatologen erfordert, da es sich um eine untersucherabhängige Methode handelt, die bestimmte Artefakte erzeugen und somit zu einer Fehlinterpretation der sonografischen Befunde führen kann.^39^

### Schlussfolgerungen

Der Bedarf an einer Methode zur sofortigen intraoperativen Bestimmung der Tumorränder, vergleichbar mit der Histologie, hat die Entwicklung und Erforschung verschiedener bildgebender Verfahren vorangetrieben. Basierend auf der vorliegenden Studie hat HFUS das Potenzial, Tumorränder sowohl in vivo als auch *ex vivo* zuverlässig zu identifizieren. Wobei der Hauptvorteil seine Verfügbarkeit, schnelle perioperative Anwendung, Zeitersparnis und die Bereitstellung essenzieller Informationen für eine optimierte chirurgische Vorgehensweise ist. Darüber hinaus könnte der routinemäßige Einsatz von HFUS im dermatochirurgischen Setting – insbesondere während der Mohs‐Chirurgie oder mikrographisch kontrollierten Chirurgie bei NMSC – die Anzahl der Eingriffe und die Hospitalisierungsdauer der Patienten signifikant reduzieren und damit die damit verbundenen Kosten (Operationssäle, Personal, pathologische Untersuchungen) senken. Dies sollte jedoch in größeren multizentrischen Studien prospektiv getestet werden. In unklaren Fällen von Hochrisikotumoren mit perineuraler Infiltration oder sehr oberflächlichen sklerodermiformen Läsionen, insbesondere in sonnenexponierten Bereichen, sollte die endgültige histologische Beurteilung abgewartet werden, bevor der Defekt verschlossen wird.

## DANKSAGUNG

Die Autoren danken Dr. Tina Weiss für die histologischen Beurteilungen und Fotografien sowie Heiko Grandel für seine Expertise in Fotografie und Formatierung.

Open access Veröffentlichung ermöglicht und organisiert durch Projekt DEAL.

## INTERESSENKONFLIKT

Keiner.
